# Antimony resistance and gene expression in *Leishmania*: spotlight on molecular and proteomic aspects

**DOI:** 10.1017/S0031182023001129

**Published:** 2024-01

**Authors:** Rajamanthrilage Kasun Madusanka, Nadira D. Karunaweera, Hermali Silva, Angamuthu Selvapandiyan

**Affiliations:** 1 Department of Parasitology, Faculty of Medicine, University of Colombo, No. 25, Kynsey Road, Colombo 8, Sri Lanka; 2 Department of Molecular Medicine, School of Interdisciplinary Sciences and Technology, Jamia Hamdard, New Delhi 110062, India

**Keywords:** antimonial, antimony resistance, gene expression, *Leishmania*, leishmaniasis, poor drug response, resistance biomarkers, treatment

## Abstract

Leishmaniasis is a vector-borne parasitic disease caused by *Leishmania* parasites with a spectrum of clinical manifestations, ranging from skin lesions to severe visceral complications. Treatment of this infection has been extremely challenging with the concurrent emergence of drug resistance. The differential gene expression and the discrepancies in protein functions contribute to the appearance of 2 distinct phenotypes: resistant and sensitive, but the current diagnostic tools fail to differentiate between them. The identification of gene expression patterns and molecular mechanisms coupled with antimony (Sb) resistance can be leveraged to prompt diagnosis and select the most effective treatment methods. The present study attempts to use comparative expression of Sb resistance-associated genes in resistant and sensitive *Leishmania*, to disclose their relative abundance in clinical or *in vitro* selected isolates to gain an understanding of the molecular mechanisms of Sb response/resistance. Data suggest that the analysis of resistance gene expression would verify the Sb resistance or susceptibility only to a certain extent; however, none of the individual expression patterns of the studied genes was diagnostic as a biomarker of Sb response of *Leishmania*. The findings highlighted will be useful in bridging the knowledge gap and discovering innovative diagnostic tools and novel therapeutic targets.

## Graphical abstract

**Figure:**
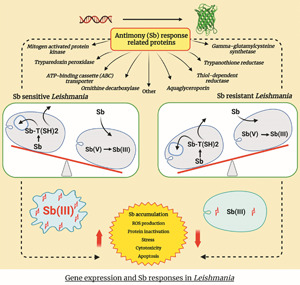

## Introduction

Leishmaniasis is a complex infectious disease caused by unicellular parasites of the genus *Leishmania* and has become a huge burden on many of the undeveloped and developing tropical countries worldwide (Desjeux, 1996; World Health Organisation, 2023). The microbial promastigote stage of this parasite is transmitted to mammalian hosts, including humans, by *Phlebotomus* or *Lutzomyia* sand flies (Killick-Kendrick, 1999; Burza *et al*., 2018). Leishmaniasis causes approximately 1–2 million cases and more than 20 000 deaths annually, while 350 million people are at risk (World Health Organization, 2010; Alvar *et al*., 2012; PAHO/WHO Leishmaniasis Fact Sheet, 2017; World Health Organisation, 2023). Impoverished people and dearth of healthcare facilities have become the major instigators of the disease, which have exacerbated the current risks of acquiring this disease to a significant level (Burza *et al*., 2018; Selvapandiyan *et al*., 2019; World Health Organisation, 2023).

There are different clinical manifestations of leishmaniasis viz. visceral (VL), cutaneous (CL), and mucosal, whereas the most life-threatening and commonly reported cases are VL and CL forms, respectively (Burza *et al*., 2018; PAHO/WHO Leishmaniasis Fact Sheet, 2017). VL, which is abundant in Africa, Brazil and India, causes severe damage to the reticuloendothelial system with dissemination of parasites, and more than 95% of cases ending up fatally, if left untreated (Aronson *et al*., 2017; World Health Organisation, 2023). About 95% of CL occur in the Americas, Asia, and the Mediterranean basin, and the majority of mucocutaneous cases are reported in countries such as Ethiopia, Bolivia, Peru, and Brazil (Burza *et al*., 2018). Thus, the widespread nature, and the potential risk of the outbreak of this disease necessitate expeditious disease control measures and prevention campaigns, along with rapid diagnosis, increased public awareness, and effective treatment strategies.

A number of different treatment strategies, like chemotherapy, thermotherapy, and cryotherapy are currently being used to treat leishmaniasis globally, but antimony (Sb) is the mainstay of treatment (Guerin *et al*., 2002; Silva *et al*., 2021*a*, 2021*b*; Madusanka *et al*., 2022). Sodium stibogluconate (SSG, pentostam) and meglumine antimoniate (MA, glucantime) are the 2 major medicaments of pentavalent Sb (Sb(V))based drugs in use (Guerin *et al*., 2002; Haldar *et al*., 2011). The most effective dosage of Sb(V) is 20 mg kg^−1^ day^−1^ for 20–28 days, and the injections may cause localized pain (Madusanka *et al*., 2022). Initially, antimonials were tremendously successful; however, the responsiveness has dwindled over the decades of use, and their current therapeutic prospects appear dim (Silva *et al*., 2021*a*, 2021*b*). The increased drug unresponsiveness in leishmaniasis is attributed to the inappropriate use of drug regimens, resulting in progressive drug tolerance in parasites (Sundar *et al*., 1994). Both the metal-containing-drug activity and the emergence of resistance in *Leishmania* are closely associated with the trypanothione-based thiol metabolism (Mukhopadhyay *et al*., 1996; Ouellette *et al*., 2004; Krauth-Siegel and Comini, 2008; Monte-Neto *et al*., 2011), and the resistance has been particularly linked to increased Sb detoxification and sequestration (Moreira *et al*., 2013; Gazanion *et al*., 2016; Dumetz *et al*., 2018). Apart from that plethora of genes, protein functions and metabolic pathways are interconnected with the arousal of Sb unresponsiveness, which is of greatest concern for its epidemiology and threatens to undermine disease control efforts. The differential gene expression and genetic modifications are of paramount importance for *Leishmania* in bringing about drug resistance, and such discrepancies are informative in predicting possible drug responses (Carter *et al*., 2006; Kumar *et al*., 2010; Torres *et al*., 2010; Biyani *et al*., 2011; Adaui *et al*., 2011*a*, 2011*b*; Oliaee *et al*., 2018; Ghosh *et al*., 2020, 2022). Often, the Sb resistance is accompanied by the transcriptional modifications of a certain set of genes that collaboratively interfere with the therapeutic effect of Sb detoxification through the incorporation of its active form into conjugates, and diminishing the intracellular Sb build up (Haimeur *et al*., 2000). For example, Patino *et al*. demonstrated the presence of more than 800 differentially expressed genes in Sb-resistant and -sensitive *Leishmania* (Patino *et al*., 2019). Moreover, a proteomic study quantitatively evaluated the Sb-resistant and -susceptible isolates of *Leishmania donovani*, whose genes were differentially expressed in relation to stress-related pathways, intracellular survival, and other key metabolic pathways (Biyani *et al*., 2011). Apart from that, studies have reported differential gene expression in Sb resistance (Walker *et al*., 2012; Das *et al*., 2015; Andrade *et al*., 2020).

Although there are a multitude of findings published on Sb resistance-related gene expression, indicating both parallel and contradictory observations, an overall discussion based on the findings of individual studies is warranted to determine the collective scientific significance. In this review, we strived to summarize the variations of Sb resistance-related gene expressions in *Leishmania* with reference to their relative abundances in terms of mRNA or protein level fluctuations. Furthermore, this study will disclose intriguing areas related to the battle against Sb resistance, which would help in navigating future research towards more productive discoveries in disease control and prevention of leishmaniasis.

## Molecular basis of Sb effect and resistance

**Figure 1.**
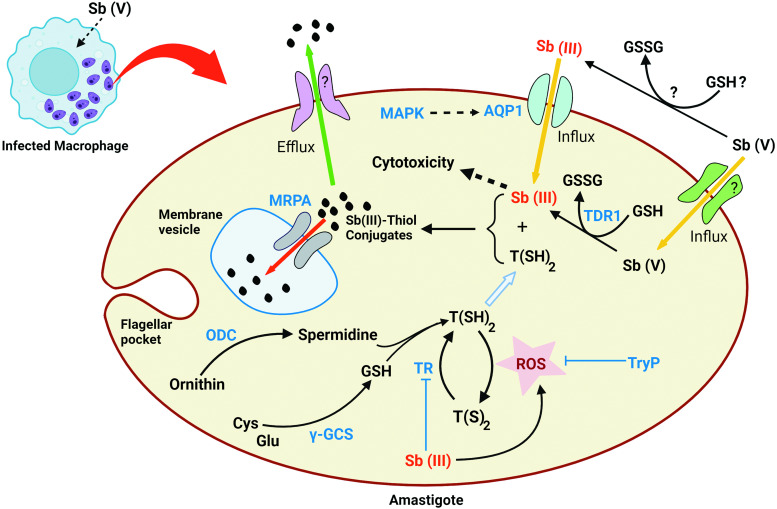
Sb metabolism and related gene expression in wild-type *Leishmania* amastigote. TR, trypanothione reductase; TDR1, thiol-dependent reductase 1; *γ*-GCS, gamma-glutamylcysteine synthetase; MRPA, multidrug-resistant protein A; AQP1, aquaglyceroporin 1; ODC, ornithine decarboxylase; TryP, tryparedoxin peroxidase; MAPK, mitogen-activated protein kinase; GSH, glutathione; T(SH)_2_, trypanothione; TS_2_, trypanothione disulphide; ROS, reactive oxygen species.

For more than 6 decades, Sb was the first line of treatment against all forms of leishmaniasis that showed high efficacy (Haldar *et al*., 2011; Negera *et al*., 2012). According to the pro-drug model, Sb(V) reduces to its active trivalent state (Sb(III)) by trypanothione (T(SH)_2_), the most effective intracellular thiol in *Leishmania* parasites (Dos Santos Ferreira *et al*., 2003), either within the host cell (López *et al*., 2015), prior to importation into the parasite, or within the parasite itself (Shaked-Mishant *et al*., 2001; Denton *et al*., 2004; Zhou *et al*., 2004; Haldar *et al*., 2011). Moreover, host macrophage thiols like glutathione (GSH) and glycylcysteine are also known to achieve non-enzymatic Sb reduction (Dos Santos Ferreira *et al*., 2003). Antimonials have been found to enter the parasite cells *via* phosphate transporters (Rosen, 2002). Most notably, this molecular reduction followed by the production of more toxic Sb(III), which exerts a lethal effect on *Leishmania*, is stage-specific as it predominantly takes place in the amastigotes compared to the promastigotes, which in turn elucidates the comparatively higher Sb(V) susceptibility of amastigotes (Callahan *et al*., 1994; Ephros *et al*., 1999; Shaked-Mishant *et al*., 2001; Goyard *et al*., 2003). The exact mechanism of the therapeutic action is an enigma, and it is believed that Sb induces parasite cell apoptosis (Fig. 1) through genomic DNA degradation, accumulation of reactive oxygen species (ROS) and nitric oxide, diminishing mitochondrial potential, and increasing intracellular Ca^2+^ (Sereno *et al*., 2001; Lee *et al*., 2002; Sudhandiran and Shaha, 2003; Basu *et al*., 2006; Vergnes *et al*., 2007; Moreira *et al*., 2011; Garg and Goyal, 2015). These impose a lethal stress on parasites by inhibiting macromolecular synthesis and energy metabolism to diminish their vital metabolic pathways, together with the interruption of glycolysis and fatty acid oxidation, which ultimately lead to the death of parasites (Berman *et al*., 1985; Herman *et al*., 1987).

## Sb resistance triggered gene expression

**Figure 2.**
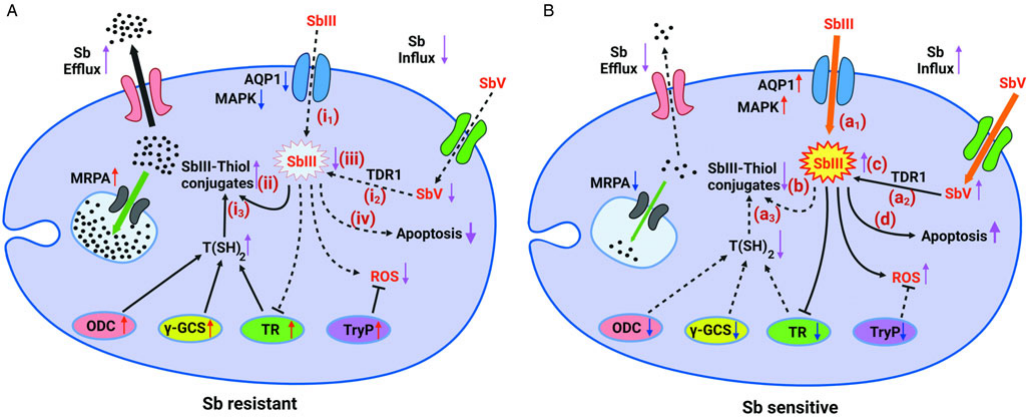
Comparison of Sb metabolism and related gene expression in Sb-resistant *vs* -sensitive amastigotes. The vertical red and blue arrows indicate gene upregulation and downregulation, respectively. The purple vertical arrows exhibit the increase/decrease of each component. TR, trypanothione reductase; TDR1, thiol-dependent reductase 1; *γ*-GCS, gamma-glutamylcysteine synthetase; MRPA, multidrug-resistant protein A; AQP1, aquaglyceroporin; ODC, ornithine decarboxylase; TryP, tryparedoxin peroxidase; MAPK, mitogen-activated protein kinase; T(SH)_2_, trypanothione; TS_2_, trypanothione disulphide; ROS, reactive oxygen species. The (i_1_), (i_2_), (i_3_), (a_1_), (a_2_), and (a_3_) are mechanisms that affect the intracellular Sb concentration (A): (i_1_) decreased Sb influx; (i_2_) decreased Sb(V) to Sb(III) conversion; (i_3_) decreased T(SH)_2_ synthesis; (ii) decreased Sb–thiol conjugate formation; (iii) increased Sb(III) accumulation; (iv) decreased cell apoptosis. (B) (a_1_) decreased Sb influx; (a_2_) increased Sb(V) to Sb(III) conversion; (a_3_) increased T(SH)_2_ synthesis; (b) increased Sb–thiol conjugate formation; (c) decreased Sb(III) accumulation; (d) increased cell apoptosis

The exact molecular mechanisms and biochemistry of Sb resistance in *Leishmania* still remain ambiguous and yet to be expounded (Fernandez-Prada *et al*., 2018). However, the mostly argued mechanism of Sb resistance is linked to the increased Sb(III) detoxification and sequestration (Ashutosh *et al*., 2007; Garg and Goyal, 2015; Gazanion *et al*., 2016), which subsequently result in reduced Sb accumulation within parasites (Fig. 2) (Ouellette and Papadopoulou, 1993; Ouellette *et al*., 2004). Furthermore, the formation of Sb–trypanothione conjugates in the presence of excess trypanothione and its rapid extrusion are largely exploited for Sb resistance in *Leishmania* (Mukhopadhyay *et al*., 1996; Rai *et al*., 2013). There were consistently high thiol levels in Sb-resistant and genetically different clinical isolates of *Leishmania* (Khanra *et al*., 2022).

Sb resistance in *Leishmania* is markedly associated with the expression of proteins related to Sb reduction, transport, and thiol synthesis (Table 1) (Khanra *et al*., 2022). Recent publications revealed 844 and 803 differentially expressed genes between Sb(SSG)-resistant and -sensitive *Leishmania braziliensis* and *Leishmania panamensis,* respectively, with over 100 genes showing ⩾2-fold change in resistant lines of each strain (Patino *et al*., 2019). Sb-resistant overexpression of transcripts assigned in the gene ontology categories such as ubiquitination, host–parasite interaction, protein phosphorylation, microtubule-based movement, and cellular process and the downregulated processes were rRNA processing, ribosome biogenesis, ribonucleoprotein complex, nucleosome assembly, and translation (Andrade *et al*., 2020). In addition, most of the differentially expressed proteins in sodium antimony gluconate (SAG)-sensitive isolate AG83-S *vs* SAG-resistant GE1-R were related to translation and metabolic enzymes (Biyani *et al*., 2011).

**Table 1.** List of Sb resistance-related genes in *Leishmania*

Gene name	Abbreviation	Functions/relevance	Expected expression in Sb-resistant *Leishmania* (upregulation/downregulation)	References
Multidrug-resistant protein A	MRPA	Sb detoxification and intracellular sequestration	Upregulation	Barrera *et al*. (2017); El Fadili *et al*. (2005); Fekrisoofiabadi *et al*. (2019); Moreira *et al.* (2013); Mukherjee *et al*. (2007)
Aquaglyceroporin 1	AQP1	Uptake of Sb(III)	Downregulation	Khanra *et al*. (2022); Mandal *et al*. (2015); Mandal *et al*. (2010); Marquis *et al.* (2005); Monte-Neto *et al*. (2015); Sharma *et al*. (2015)
Gamma-glutamylcysteine synthetase	*γ*-GCS	Thiol biosynthesis	Upregulation	Carter *et al*. (2006); Fonseca *et al*. (2017); González-Chávez *et al*. (2019); Grondin *et al*. (1997); Mukherjee *et al*. (2007)
Mitogen-activated protein kinase 1	MAPK1	Signal transduction cellular stress response, proliferation, infectivity, differentiation and apoptosis	Downregulation	Garg and Goyal (2015); Leonard *et al*. (2004); Mann *et al*. (2006)
Ornithine decarboxylase	ODC	Spermidine biosynthesis	Upregulation	Fonseca *et al.* (2017); Gómez Pérez *et al*. (2016); Haimeur *et al.* (1999); Mukherjee *et al*. (2007)
Trypanothione reductase	TR	Reduction of trypanothione	Upregulation	Ghosh *et al*. (2017); Krauth-Siegel and Inhoff (2003); Nateghi-Rostami *et al*. (2022); Zabala-Peñafiel *et al*. (2023)
Thiol-dependent reductase 1	TDR1	Reduction of Sb(V) to Sb(III)	Upregulation	Denton *et al*. (2004); Nateghi-Rostami *et al*. (2022)
Tryparedoxin peroxidase	TryP	Detoxification of harmful peroxides	Upregulation	Andrade and Murta (2014); Das *et al*. (2018); González-Chávez *et al*. (2019); Iyer *et al*. (2008); Nateghi-Rostami *et al*. (2022); Wyllie *et al*. (2008, 2010)
Heat shock protein-60	HSP60	Unknown	Unknown	Matrangolo *et al.* (2013); Peláez *et al*. (2012)
Heat shock protein-70	HSP70	Unknown	Unknown	Brochu *et al*. (2004); Özbilgin *et al.* (2021); Peláez *et al.* (2012)
Heat shock protein-83	HSP83	Unknown	Unknown	Kumar *et al.* (2012); Matrangolo *et al.* (2013)
Histone 1	H1	Linker protein of nucleosome	Unknown	Martínez *et al.* (2002); Fasel *et al.* (1993); Singh *et al.* (2010)
Histone 2A	H2A	Core histone of nucleosome	Unknown	Abanades *et al.* (2009); Singh *et al*. (2010); Soto *et al*. (1992)
Histone 4	H4	Core histone of nucleosome	Unknown	Kumar *et al.* (2012); Soto *et al*. (1997)
Antimony resistance marker 56	ARM56	Unknown	Unknown	Rugani *et al*. (2019)
Antimony resistance marker 58	ARM58	Unknown	Unknown	Nühs *et al*. (2014); Rugani *et al*. (2019); Schäfer *et al*. (2014)
Pentamidine resistance protein 1	PRP1	Unknown	Unknown	Coelho *et al.* (2003); Khanra *et al*. (2022)
Parasite surface antigen-2	PSA-2	Immunogenic antigen	Unknown	Bhandari *et al.* (2013)
Arsenate reductase-2	ACR2	Reduction of Sb(V) to Sb(III)	Upregulation	Khanra *et al.* (2022); Zhou *et al.* (2004)

Copy number variation (CNV) is among the crucial molecular mechanisms deployed by *Leishmania* to increase the transcript levels of resistance genes. CNV involves the duplication of either specific genomic region or complete chromosomes as intra- or extrachromosomal elements (Ullman, 1995; Leprohon *et al*., 2015; Papadopoulou *et al*., 2016). Moreover, gene amplification that occurs in a part of the amplicon or intrachromosomal level has also been observed in differential gene expression, which leads to metal-drug resistance in *Leishmania* including Sb stress (Grondin *et al*., 1997; Leprohon *et al*., 2009; Mukherjee *et al*., 2013). The presence of single-nucleotide polymorphisms (SNPs) in genes encoding functional and structural proteins related to Sb resistance is known to regulate *Leishmania* resistance towards chemotherapy (Downing *et al*., 2011; Coelho *et al*., 2012; Rastrojo *et al*., 2018). The presence of 3 SNPs in serine acetyltransferase, the protein involved in cysteine synthesis, exhibited increased Sb resistance in *Leishmania infantum* mutants having impaired adenosine triphosphate (ATP)-binding cassette (ABC) transporters (Douanne *et al*., 2020). Further, an SNP that occurred in protein kinase in *L. infantum* has an influence on Sb resistance (Brotherton *et al*., 2013). The resistance phenotype of *Leishmania* is a final product of various cellular mechanisms, including gene overexpression (supplementary material) accompanied by preadaptations like structural and functional modulations.

## ABC transporter (multidrug-resistant protein A)

The ABC transporter superfamily consists of functionally Sb-resistant proteins of *Leishmania* that have exerted an Sb detoxification potential *via* direct membrane efflux of Sb to the extracellular milieu (El Fadili *et al*., 2005; Singh *et al*., 2014; Douanne *et al*., 2020). Multidrug-resistant protein A (MRPA, also known as ABCC3) is one of the ABC transporters, formerly identified as a P-glycoprotein (Ouellette *et al*., 1998), and has been reported to be a *Leishmania* intracellular protein found in membrane vesicles near the flagellar pocket, at the sites of endo- and exocytosis of the parasite (Fig. 1) (El Fadili *et al*., 2005; Ashutosh *et al*., 2007). Previous literature has amply demonstrated the inevitable role of MRPA in Sb resistance *via* intracellular sequestration of Sb–thiol conjugates into vesicles (Mukherjee *et al*., 2007; Moreira *et al*., 2013; Singh *et al*., 2014; Gazanion *et al*., 2016). In addition, MRPA expression resulted in an increased drug resistance in *L. donovani* in relation to altered fluidity in the cell membranes and decreased drug accumulation (Bhandari *et al*., 2014). Previously, MRPA was suggested as one of the 2 prediction models for determining Sb treatment failure that could predict the treatment outcome with high accuracy (Torres *et al*., 2013). Hence, MRPA expression could be a crucial characteristic of Sb resistance (Fig. 2). Interestingly, *L. donovani* was able to develop SSG resistance even under arsenic (As) stress because both Sb and As pressures could trigger the same overexpression of the ABC transporter (Perry *et al*., 2013).

Extrachromosomal amplification within circular amplicons of MRPA has been extensively studied in different *Leishmania* species (Mukherjee *et al*., 2007; Leprohon *et al*., 2009; Moreira *et al*., 2013). Likewise, the adaptive gene amplification of MRPA observed in *L. infantum* during *in vitro* Sb(III) selection corroborates its significance in Sb tolerance (Ubeda *et al*., 2014). More importantly, MRPA amplification confers the first line of defence against Sb(III) stress in *Leishmania*, providing the driving force for the inception of underlying molecular adaptations upon an infection and signalling pathways (Dumetz *et al*., 2018). Therefore, its expression level during drug pressure could be a determinant of the parasites' destiny as well as providing strong insights upon the subsequent development of Sb resistance. Beyond Sb transportation, an indirect correlation was explored between *Leishmania* MRPA expression and cellular redox homoeostasis that was affected by glucose-6-phosphate dehydrogenase and trypanothione reductase (TR) interaction upon metalloid exposure, including Sb (Ghosh *et al*., 2017). Dumetz *et al*. demonstrated the importance of overexpression of the H locus, which harbours the MRPA gene, over M locus and increases the Sb resistance in 3-fold (Dumetz *et al*., 2018).

MRPA was expressed in *Leishmania* with a direct correlation to Sb-resistant phenotype, and it was widely expressed in most of the parasite and clinical forms of leishmaniasis (Mukherjee *et al*., 2007; Barrera *et al*., 2017; Fekrisoofiabadi *et al*., 2019). For example, the MRPA expression was found to be markedly augmented in Sb-resistant Indian isolates of *L. donovani* and *Leishmania tropica,* respectively, compared to their sensitive counterparts (Khanra *et al*., 2022). Furthermore, 2 independent methods viz. cDNA-amplified fragment length polymorphism approach and quantitative polymerase chain reaction (qPCR) analysis demonstrated approximately similarly augmented expressions (2–3-fold) of MRPA in Sb-resistant clinical anthroponotic cutaneous leishmaniasis (ACL) isolates of *L. tropica* compared to the sensitive counterparts (Kazemi-Rad *et al*., 2013; Mohebali *et al*., 2019). An antibody assay recognized high MRPA levels in *Leishmania guyanensis* and *Leishmania amazonensis*-resistant lines, but the detection was not successful for the sensitive lines, which further demonstrated differential MRPA expression between 2 phenotypes (Moreira *et al*., 2013). Moreover, a full genome microarray hybridization in *L. amazonensis* showed a robust (5-fold) MRPA expression in Sb-resistant parasites compared to its wild-type (Monte-Neto *et al*., 2011). Interestingly, the obstruction of MRPA expression conferred increased drug susceptibilities in *Leishmania*; for instance, the MRPA null mutants of *L. infantum* promastigotes exhibited drastic declines (20-fold) in their half maximal inhibitory concentration (IC_50_) values against Sb(III), whereas the corresponding amastigotes showed increased sensitivity to Sb(V) compared to their wild-type (Douanne *et al*., 2020). Appropriately, following the selection of Sb(III) resistance, the transcript levels of sensitive parasites in a study were elevated, reaching a 1.5–3.0-fold expression as same as the resistant parasites (Dumetz *et al*., 2018). This observation affirms that the selective drug pressure is able to provoke Sb resistance in *Leishmania* through MRPA-mediated mechanisms as a preadaptation of parasites to harsh conditions, thus, it is salient to indicate that the overexpression of this protein may enable the parasites to withstand or weaken the Sb therapeutic effect as a successful counter mechanism. In addition, studies have reported adaptive expression of MRPA in an Sb concentration-dependent manner, whereas an initial elevation of the copy number was seen in all the Sb(III)-resistant mutants, and it was gradually decreased to the wild-type level in the subsequent several passages in the absence of drug pressure (Haimeur *et al*., 2000). This would further inform the drug pressure-induced overexpression and the significance of MRPA-mediated pathways to achieve Sb resistance in certain instances.

Contrariwise, MRPA-independent resistance mechanisms were also possible, in which it was not ubiquitously upregulated in all the Sb-resistant isolates of a study (Moreira *et al*., 1998; Mukherjee *et al*., 2007). Accordingly, MRPA-independent Sb resistance was accompanied by unchanged mRNA levels in resistant *Leishmania*, whereas it was sometimes considered a protein without an important role in Sb transportation (Dos Reis *et al*., 2017) or a non-essential transcript for Sb resistance; however, the disruption of this protein triggered Sb hypersensitivity in both amastigotes and promastigotes of *L. infantum* (Douanne *et al*., 2020). The presence of a high resistance index was found to be essential for the upregulation of MRPA, while the energy-dependent Sb resistance pathway of resistant mutants did not rely on the upregulation of this gene (Rai *et al*., 2013; Dos Reis *et al*., 2017). In a study related to *L. donovani*, Kumar *et al*. observed upregulation of MRPA in 6 resistant and 1 sensitive isolate with no significant elevation of its expression in another 4 resistant isolates of the same species (Kumar *et al*., 2012). Moreover, the MRPA level of *L. panamensis* was augmented only in *in vitro*-adapted Sb-resistant strains, and no significant difference was observed in clinically resistant lines (Barrera *et al*., 2017). This was further corroborated by the studies that have reported no or negligible amplification of MRPA in glucantime-resistant clinical *Leishmania* isolates (Ullman *et al*., 1989; Moreira *et al*., 1998; Gómez Pérez *et al*., 2016). In addition, an MRPA amplification was observed only in 3 out of 4 SAG-resistant isolates, and the rest did not show any sign of amplification (Mukherjee *et al*., 2007). Therefore, there must be multiple factors behind the MRPA expression under Sb exposure that can modulate its expression. Accordingly, MRPA was a candidate marker for drug resistance with 69% accuracy as a prediction model in determining the treatment outcome of clinical Sb-treatment upon *L. braziliensis* (CL) (Torres *et al*., 2013).

Of note, many studies have surveyed paradoxical results about MRPA expression linked to its expression in Sb-resistant and -sensitive parasites, which are not satisfactorily resolved yet. Although MRPA seemed to have wide expression in *Leishmania*, species-specific discrepancies of the expression levels were also possible in Sb resistance. For example, a quantitative reverse transcription (RT)-PCR analysis showed no differential expression in Sb-resistant *L. infantum*, in spite of 2-fold increased mRNA expression in Sb(III)-resistant isolates of *L. guyanensis*, *L. braziliensis,* and *L.* amazonensis compared to the susceptible lines (Moreira *et al*., 2013). Moreover, increased MRPA levels were seen (1.5–25.2-fold) in Sb(III)-resistant *L. braziliensis* and Sb(V)-unresponsive *L. tropica* along with simultaneous expression in respective responsive lines, so it diminishes the possibility of the functional relevance of MRPA in resistance phenotypes (Oliaee *et al*., 2018; Rugani *et al*., 2019). On the other hand, Victoria *et al*. demonstrated that sitamaquine can successfully circumvent the Sb resistance caused by MRPA expression (Pérez-Victoria *et al*., 2011), and the MRPA-mediated Sb resistance was reverted by buthionine sulphoximine, a GSH biosynthesis-specific inhibitor (El Fadili *et al*., 2005). Hence, the obstruction of the interaction between MRPA and GSH may be an effective approach for drug design (Fekrisoofiabadi *et al*., 2019).

Moreover, Callahan *et al*. demonstrated an oxidative state-dependent selective Sb resistance in *Leishmania major*, where the MRPA expression could manifest resistance to Sb(III) and not against Sb(V), albeit with clear evidence for the intracellular conversion of Sb(V) to its reduced form during its mode of action in the parasites (Callahan and Beverley, 1991; Dos Santos Ferreira *et al*., 2003). Moreover, significant MRPA expression was observed in promastigotes under increased Sb(III) stress compared to the intracellular amastigotes exposed to Sb(V) (Gazanion *et al*., 2016; Fernandez-Prada *et al*., 2018). Accordingly, the amastigotes must have evolved pathways to confer drug protection *via* MRPA-independent mechanisms as well. Collectively, the MRPA expression in Sb resistance is not a consistent event, which is broadly affected by multiple factors.

## Aquaglyceroporin (AQP1)

Aquaglyceroporins are a subcategory of aquaporins that primarily involve water and glycerol transportation in mammalian cells (Verkman, 2008; Mukhopadhyay *et al*., 2014). In *Leishmania*, AQP1 has been implicated as a protein that imports Sb(III) into the cells, and its decreased expression has been broadly discussed and attributed to Sb resistance in many studies (Fig. 2A) (Gourbal *et al*., 2004; Gómez Pérez *et al*., 2016). It is predominantly found in the flagellums of the promastigote stage, which is then relocated to the parasite surface eventually after post-translational phosphorylation by the mitogen-activated protein kinase (MAPK) (Mandal *et al*., 2012; Sharma *et al*., 2015). Since the AQP1 expression is highly associated with the Sb accumulation in *Leishmania*, reduced expression or the perturbation of its gene expression has been extensively reported in relation to Sb resistance (Gourbal *et al*., 2004; Marquis *et al*., 2005; Mukherjee *et al*., 2013; Sharma *et al*., 2015; Mohebali *et al*., 2019; Khanra *et al*., 2022).

AQP1 expression increased the Sb(III) accumulation in *Leishmania* compared to the untreated control (Sharma *et al*., 2015); therefore, the suppression of its transcripts is much preferred by the resistant *Leishmania* and vice versa (Fig. 2) (Kazemi-Rad *et al*., 2013; Douanne *et al*., 2020). Accordingly, single-allele disruption or subtelomeric deletion of AQP1 resulted in drastically reduced Sb accumulation in *Leishmania,* accompanied by prompt Sb resistance (Gourbal *et al*., 2004; Monte-Neto *et al*., 2015). Furthermore, terminally deleted mutants of AQP1 could restore their Sb(III) resistance following the episomal transfection of the gene, through which the IC_50_ of the mutants (by 20–50-fold) subsequently dropped in a rigorous decline (Mukherjee *et al*., 2013). The AQP1 expression in Sb-resistant isolates of ACL showed greater suppression than that of the sensitive strains (Kazemi-Rad *et al*., 2013), and more significantly, a negative correlation was seen between AQP1 expression and the IC_50_ or time taken to cure ACL lesions in the responsive cases of natural isolates (Oliaee *et al*., 2018; Khanra *et al*., 2022). Similarly, the decreased expression of AQP1 in the Sb-resistant clinical isolates of *L. donovani* and *L. tropica* was further affirmed by a negative correlation between IC_50_ and AQP1 expression (Mohebali *et al*., 2019; Khanra *et al*., 2022). *Leishmania* parasites of CL or post kala-azar dermal leishmaniasis (PKDL) had more robust antimonial accumulation than that of the VL and were more Sb-sensitive, which was rendered so by the elevated expression and mRNA stability of AQP1 (Mishra *et al*., 2013; Mandal *et al*., 2015). A downregulation of AQP1 was seen in the majority of Sb(III)-resistant VL and PKDL-derived *L. donovani* isolates albeit with several exceptions (Mandal *et al*., 2010). Not only that, an atypical form of tegumentary leishmaniasis caused by *L. braziliensis* showed an outstanding 65-fold downregulation of AQP1 in their clinical isolates than that of the reference strain (Rugani *et al*., 2019). In addition, there was a comparable AQP1 expression in the Sb treatment failure isolates of *L. major,* which was found to be 58.71-fold less than that of the treatment-responsive isolates (Sharma *et al*., 2015). More importantly, the 2 Sb transporters, MRPA and pentamidine resistance protein 1 (PRP1), were found to have increased their expression in clinically resistant parasites along with simultaneous suppression of AQP1 transcripts, with an emphasis on the increased Sb detoxification plus decreased influx (Khanra *et al*., 2022). Apart from that, the AQP1 expression in Sb(V)-resistant *L. donovani* isolated from Nepal and the AQP1 copy number derived from chromosome 31 in the resistant mutants of *L. major* were found to be lower than that of their sensitive strains (Decuypere *et al*., 2005). Interestingly, the transfection of AQP1 followed by its increased expression in *Leishmania tarentolae*, *L. major,* and *L. infantum* developed hypersensitivity to metalloids such as As(III) and Sb(III) (Gourbal *et al*., 2004). Another study also revealed supportive evidence of similar hypersensitivity in *L. major* isolates of CL patients, and secondarily, their resistance emerged with the deletion, inactivation through mutation and reduced expression of AQP1 (Eslami *et al*., 2021). Conversely, *in vitro* transfection failed to enhance Sb susceptibility of resistant promastigote lines as well as sensitive lines compared to their respective parent strains (Mandal *et al*., 2010). *Leishmania donovani* clinical isolates of SAG-resistant and -sensitive parasites showed marked down- and upregulations respectively, whereas the expression difference was more prominent between the amastigote lines than the respective promastigotes. The Sb(V)-resistant *L. donovani* isolated from Nepal exhibited 6–7-fold significantly lower AQP1 expression than that in the sensitive strains (Decuypere *et al*., 2005).

There are many disputes among research findings about the AQP1 expression in drug-resistant *Leishmania*. For instance, Maharjan *et al*. suggested that the downregulation of AQP1 was just one of the Sb-resistant mechanisms in *Leishmania* and that not all the resistant ones consistently downregulate it (Maharjan *et al*., 2008). Further, it was ascertained by the high AQP1 copy number observed in the resistant parasites compared to the sensitive ones, which was not in line with the reduced import of Sb(III) (Maharjan *et al*., 2008). It was also suggested that AQP1 is not an essential protein for the survival of *Leishmania* (Plourde *et al*., 2015). *In vitro*-selected Sb-resistant mutants *L. braziliensis*, *L. infantum,* and *L. guyanensis* did not show a significant difference in AQP1 mRNA level compared to the control, in agreement with the absence of its function in Sb transportation in non-natural-resistant mutants (Torres *et al*., 2010; Moreira *et al*., 2013; Dos Reis *et al*., 2017). Interestingly, a study on *L. panamensis* revealed decreased AQP1 levels of *in vitro*-adapted Sb-resistant strains, and no significant difference was observed in the clinically resistant lines (Barrera *et al*., 2017). Therefore, growing evidence has suggested the possibility of AQP1 neutral Sb resistance, and therefore, it has also hinted at the prevalence of many critical cellular functions of these transcripts other than the Sb influx.

In agreement with the wide array of functions achieved by the AQP1, a handful of reports indicate its upregulation without affecting the inherited Sb resistance and noticeably suggest an alternative mechanism of Sb resistance. *Leishmania infantum* amastigotes with Sb(III) resistance had increased AQP1 expression, which was reverted to the wild-type in the presence of drug pressure (Marquis *et al*., 2005). Further, *L. major* parasites isolated from non-healing cases showed increased AQP1 expression (Eslami *et al*., 2016; Alijani *et al*., 2019). In a study aiming to investigate the biomarkers of Sb resistance, *L. donovani* showed a marked AQP1 upregulation in all the selected clinically Sb-sensitive isolates, in comparison to significant downregulation observed in only 30% of resistant ones, whereas others showed similar expression to the wild-type (Kumar *et al*., 2012). According to the available evidence on AQP1 expression, it may be a multifunctional protein in *Leishmania* that is also significantly involved in Sb resistance. Therefore, many elaborate studies are warranted to clearly understand the network of those functions.

## Gamma-glutamylcysteine synthetase

Gamma-glutamylcysteine synthetase (*γ*-GCS, l-glutamate: l-cysteine *γ*-ligase) catalyses the rate-limiting step of GSH biosynthesis that leads to the trypanothione overexpression (Mukhopadhyay *et al*., 1996; Haimeur *et al*., 2000; Lu, 2001). During its mode of action, firstly, *γ*-GCS triggers the covalent bond formation between glutamate and cysteine to synthesize gamma-glutamylcysteine, which in turn binds with glycine, resulting in GSH formation (Fig. 1) (Olin-Sandoval *et al*., 2012). Accordingly, the activity of this protein is controlled by the intracellular GSH levels, and the non-allosteric feedback, as well as the transcriptional and translational factors (Lu, 2001). Moreover, *γ*-GCS overexpression was considered to confer increased virulence, cell viability, and drug resistance in parasites (Pérez-Rosado *et al*., 2002; González-Chávez *et al*., 2019).

The *γ*-GCS has been reported to be upregulated in Sb-resistant *Leishmania* and implicated as a protein that triggers Sb-detoxification pathways (Fig. 2A) (Grondin *et al*., 1997; Mukherjee *et al*., 2007). For example, an elevated *γ*-GCS expression was observed in therapeutic failure in *L. guyanensis* in all the *in vitro* growth phases of the promastigote (Torres *et al*., 2010). Its expression in Sb-resistant *L. major* derived from CL patients was 20 times higher than that of the sensitive and it was suggested to be a possible biomarker in the identification of clinical resistance (Ghobakhloo *et al*., 2016). Fittingly, there was a positive correlation between *γ*-GCS expression and the IC_50_ values of Sb-resistant clinical kala-azar isolates of *L. tropica* and *L. donovani* with several-fold overexpression (Khanra *et al*., 2022). The *γ*-GCS was not merely associated with developing Sb resistance; however, its depleted expression was associated with adverse effects on parasites; for instance, the downregulation of these transcripts rendered decreased parasite oxidative defence that made the parasites more susceptible to drug effects, which was in line with the reported upregulation and downregulation in the majority of resistant isolates and the sensitive ones, respectively (Fig. 2). The RNA expression level of *γ*-GCS was 2.1 times higher in clinical Sb-resistant isolates of *L. tropica* compared to the sensitive isolates, but it was upregulated only in 70% of resistant isolates, whereas 75% of sensitive isolates experienced downregulations (Kumar *et al*., 2012). Additionally, *γ*-GCS expression was dependent on the host organ and the type of *Leishmania* strain, which informs about the influence of environmental factors that could govern its expression (Carter *et al*., 2006). Apart from that, *γ*-GCS expression has been attributed to rapid wound healing in ACL, which was ascertained by a negative correlation seen between *γ*-GCS expression and the time taken to cure lesions of the responsive cases of field isolates (Oliaee *et al*., 2018). Hence, this implicates the functional relevance of *γ*-GCS expression with a possible relation to GSH-dependent pathways to accelerate the healing process.

There were also discrepancies in *γ*-GCS expression levels in *Leishmania* in relation to Sb stress and parasite defence. The resistant *Leishmania* isolates, including the resistant standards, were neutral in *γ*-GCS expression, while the sensitive parasites showed inconsistencies of expression having either been upregulated (2.32-fold), downregulated (<0.6-fold), or unaltered (Mohebali *et al*., 2019). Rai *et al*. suggested that *γ*-GCS is not consistently expressed, is not involved in naturally Sb-resistant *Leishmania,* or has a role only in highly resistant parasites (Rai *et al*., 2013). Furthermore, a pronounced downregulation was seen in Sb-resistant *L. donovani* (Decuypere *et al*., 2005, 2008). Even though *γ*-GCS has been studied as an inducer of thiol biosynthesis, *γ*-GCS-independent thiol elevations have also been characterized in natural Sb-resistant *L. donovani*. Furthermore, the *γ*-GCS amplification was found to be negligible in those parasites, showing that it was not directly involved in the thiol synthesis of that particular strain (Mittal *et al*., 2007). Based on the current evidence, thiol production may not be solely dependent on the *γ*-GCS activity, which may sometimes enable its differences in expression without interfering with Sb resistance, thus minimizing the likelihood of this protein being a potential expression marker of Sb resistance.

## Mitogen-activated protein kinase

MAPKs are primarily involved in the phosphorylation of other proteins and are associated with cellular stress response, proliferation, infectivity, differentiation, and apoptosis (Wiese, 1998; Hindley and Kolch, 2002). There are around 17 different MAPK proteins in *Leishmania.* MAPK3 and MAPK9 are exclusively expressed in the promastigote stage and are involved in flagellum maintenance (Bengs *et al*., 2005), whereas MAPK1 and MAPK2 are implicated in Sb resistance (Sharma *et al*., 2015). The Sb resistance achieved through MAPK was found to have been associated with the modulation of AQP activity (Mandal *et al*., 2012). Furthermore, the co-expression of MAPK with AQP1 increases Sb(III) uptake and drug sensitivity in *L. major* (Mandal *et al*., 2012). Therefore, MAPK must have at least an indirect effect on the Sb transportation mechanisms of *Leishmania* cells (Fig. 1). Metal-based drugs like Sb(III) induce ROS production leading to subsequent cell apoptosis interconnected with activation of MAPK signalling cascade (Leonard *et al*., 2004; Mann *et al*., 2006; Garg and Goyal, 2015), which is why favourable downregulation of MAPK could be a promising adaptation to avert Sb cytotoxicity.

A several-fold decreased expression of MAPK1 was observed in Sb-resistant *L. donovani* compared to the sensitive reference that showed a slight increase, which is suggestive of the possible involvement of MAPK1 in triggering cell death pathways upon Sb exposure (Ashutosh *et al*., 2012). Furthermore, observation of reduced protein levels in those resistant strains further validated the aforementioned downregulation, and besides, the MAPK overexpression enabled cells to have 2–3-fold increased susceptibility to both Sb(V) and Sb(III) than the cells transfected with the empty vectors (Ashutosh *et al*., 2012). In addition, RT-PCR assays revealed a differential expression of MAPK with a suppression of its transcript levels in Sb-resistant *L. major* and *L. tropica* clinical isolates compared to the respective sensitive parasites (Kazemi-Rad *et al*., 2013; Sharma *et al*., 2015). The deletion of MAPK2 in *L. major* resulted in reduced uptake of Sb(III) and slower healing (Mandal *et al*., 2012) because of increased parasite viability following less Sb toxicity. Contrarily, MAPK transcripts were more abundantly expressed in 90% of SAG-resistant *L. donovani* clinical isolates, together with one of the sensitive lines (Kumar *et al*., 2012). Altogether, most of the time MAPK can show decreased expression as a preadaptation to Sb resistance, but the inconsistency of its abundance could be a negative factor for the suitability of this protein as a biological Sb marker. However, the positive regulation of MAPK accompanied by AQP1-mediated Sb accumulation would be an attractive phenomenon for drug designing (Fig. 2B) (Mandal *et al*., 2012).

## Ornithine decarboxylase

Ornithine decarboxylase (ODC) is the rate-limiting protein of the polyamine biosynthetic pathway, which is important for cell growth and proliferation, and its expression is mostly mediated by gene amplification (Haimeur *et al*., 1999; Ilari *et al*., 2015). It is involved in spermidine biosynthesis as the final product (Fig. 1), and studies have revealed pronounced amplification attributed to both the Sb and As resistance in *Leishmania* (Haimeur *et al*., 1999; Fonseca *et al*., 2017). Thus, the elevated ODC may functionally assist the parasites to alleviate the antiproliferative effect caused by metalloid drugs.

There was an overexpression of ODC in Sb-resistant *L. donovani* compared to the sensitive line; however, it was not expressed on extrachromosomal circles. Apart from that, it was further validated by the protein level overexpression in all the resistant isolates (Mukherjee *et al*., 2007). Both the promastigotes and the amastigotes of *L. donovani* overexpressed their ODC levels as a self-protective mechanism against SAG, resulting in notable rises in their IC_50_ values than that of the wild-type strains (Singh *et al*., 2007). Moreover, the gene transfection followed by ODC expression was able to increase Sb(III) resistance in 2-fold compared to the wild-type parasites or empty vector-transfected *L. guyanensis*. In fact, the parasites were more susceptible to the Sb effect with the inhibition of ODC, and the opposite was experienced with the overexpression. For example, α-difluoromethylornithine (DMFO) pre-treated *L. guyanensis* cells exhibited 648-fold susceptibility to Sb(III) for wild-type in comparison to the 1.5-fold observed in the ODC-transfected clones (Fonseca *et al*., 2017). More remarkably, the canine-infected *L. infantum* clinical isolates showed increased RNA expression in Sb-resistant lines compared to the susceptible, especially in the absence of *γ*-GCS and trypanothione synthetase (TryS) expression, indicating the functional relevance of ODC in elevating T[SH]_2_ levels in resistant lines (Gómez Pérez *et al*., 2016). There was a noticeable difference in ODC gene expression between the natural resistance and resistant mutants since only the naturally resistant *L. donovani* parasites could augment the expression, while the mutants remained unchanged compared to the reference Dd8 strain (Rai *et al*., 2013). Furthermore, the ODC expression was amplified in both genetic and protein levels in Sb-resistant Indian *L. donovani* and Peruvian *L. braziliensis*, and on the contrary, a downregulation was observed in *L. donovani* isolates from Nepal (Decuypere *et al*., 2005; Mukherjee *et al*., 2007; Adaui *et al*., 2011*a*, 2011*b*), hence permitting queries about the exact biological role of this protein in Sb resistance.

Moreover, in *L. panamensis*, ODC failed to provoke Sb(III) resistance in laboratory-selected stains, owing to the possible hindrance of expression due to the activation of alternative polyamine synthesis pathways or the import of polyamines (Goyeneche-Patino *et al*., 2008). The intracellular amastigotes of clinical isolates showed strikingly decreased ODC expression in Sb(V)-resistant lines than that of the sensitive ones (Decuypere *et al*., 2005) which resulted due to the changes in ODC-mediated thiol-biosynthesis in such a way that facilitates parasite-friendly intracellular environment and arrested activation of the Sb(V) in amastigotes (Decuypere *et al*., 2005). Supporting evidence was published on Sb-resistant *L. guyanensis* mutants, whose ODC level had no relative difference compared to the parental strain (Dos Reis *et al*., 2017). The prevalence of contradictory evidence of expressions related to Sb resistance raises doubt on the diagnostic use of ODC. Accordingly, previous analysis based on Youden's *J* statistics justified the foregoing results with the observation of only 50% specificity of ODC to detect the Sb(V) resistance in *L. braziliensis* clinical isolates (Adaui *et al*., 2011*a*, 2011*b*).

## Trypanothione reductase

TR and thioredoxin peroxidase are among the several proteins involved in spermidine metabolism (Ilari *et al*., 2015), and it has become an attractive drug target since its unavailability in mammals (Krauth-Siegel and Inhoff, 2003; Vázquez *et al*., 2017). The protozoans have evolved a trypanothione/TR system instead of a GSH/glutathione reductase system that is found in mammalian cells (Fig. 1) (Baiocco *et al*., 2009). Trypanothione forms a complex with Sb(III) and it is efficiently transported *via* plasma membrane vesicles *via* an ATP-coupled efflux pump, resulting in Sb resistance (Mukhopadhyay *et al*., 1996; Gómez Pérez *et al*., 2016). In a study, TR conferred oxidative protection to both amastigotes and promastigote stages against Sb(V) and Sb(III) and consequently intensified the treatment failure capacity of the non-responders (Zabala-Peñafiel *et al*., 2023). Hence, it is rational that Sb(III) inhibits TR in a reversible manner so as to secondarily avert the reduction of trypanothione, leading to the accumulation of more disulphides, which would ultimately weaken the resistance (Fig. 2B) (Wyllie *et al*., 2004). Research findings corroborate the Sb(III)-mediated inhibition of TR in *Leishmania*, which eventually boosts the concentration of the disulphide forms of the intracellular trypanothione and glutathione, which perturbs the cellular thiol redox potential (Wyllie *et al*., 2004). For instance, inhibition of TR leads to apoptotic death of *Leishmania* parasites owing to instantaneous decline of thiol content (Ghosh *et al*., 2017), thus, TR expression is of greater importance for protozoans, not only during drug pressure but also for their survival in any circumstance.

The TR protein augmentation was proportional to its activity, with a more than doubled mean activity in non-responders *vs* responders, in the clinical isolates of *L. tropica* from ACL patients (Nateghi-Rostami *et al*., 2022). The high thiol levels observed in natural Sb-resistant *L. donovani* cells with concurrent amplification of TR and MRPA were not mediated by the *γ*-GCS (Mittal *et al*., 2007). Furthermore, a western blot analysis revealed 4-fold overexpression of TR in Sb(III)-resistant natural canine isolates of *L. infantum vs* sensitive (Gómez Pérez *et al*., 2016). The TR expression seemed to be modulated during promastigote growth, and however, it was upregulated in most of the parasites of Sb-unresponsive isolates (9/10) along with some cured ones (3/11) (Adaui *et al*., 2011*a*, 2011*b*), and it pointed out a functionally irrelevant outcome.

Additionally, the TR expression was almost the same in between the Sb-unresponsive and -sensitive *L. donovani* clinical extracts, and it was suggested that TR had no clear role in Sb resistance in *Leishmania* (Nateghi-Rostami *et al*., 2022). Studies conducted by Wyllie *et al*. did not show any correlation between the TR activity and the clinical Sb resistance in *L. donovani* isolates, which is why they suggested its negligible involvement in resistance phenotype (Wyllie *et al*., 2010). Likewise, TR showed a similar and consistently expressed pattern in both MA-responsive and -unresponsive *L. tropica*, whereas approximately 20% of samples from both types, TR was not affected by Sb (Oliaee *et al*., 2018). In summary, TR is an extremely important protein in Sb-resistant *Leishmania,* with mostly elevated expressions despite possible outliers that could diminish its functional relevance.

## Thiol-dependent reductase 1

Thiol-dependent reductase 1 (TDR1) is a tetramer with a functional domain containing omega class glutathione-*S* transferases that involves the reduction of Sb(V) to Sb(III), with the help of GSH as the reductant (Fig. 1) (Denton *et al*., 2004; Haldar *et al*., 2011). TDR1 was strikingly upregulated in the amastigotes than that of the promastigotes, and it was attributed to the TDR1-mediated reduction of Sb(V) to Sb(III), whereas promastigotes were prominently sensitive to Sb(V) than the amastigotes (Shaked-Mishant *et al*., 2001; Denton *et al*., 2004). All the MA non-responders in a study showed significantly elevated metabolic activity against hydrogen peroxide (H_2_O_2_) compared to the lower activity seen in responders, which was in line with the elevated trypanothione peroxidase levels in non-responders (Nateghi-Rostami *et al*., 2022). A 3–4-fold increased TDR expression was experienced in SSG-resistant parasites of *L. donovani* and *L. tropica* than in the sensitive line (Khanra *et al*., 2022).

Interestingly, the TDR1 expression was broadly variable in the *L. tropica* Sb-responsive isolates, with the majority of them having fold changes between 2.3 and 1124. In the meantime, the expression was not very prominent in the unresponsive lines, having high expression only in several isolates (Oliaee *et al*., 2018). Moreover, the TDR1 expression was several-fold downregulated in MA-unresponsive clinical *L. tropica* (Oliaee *et al*., 2018).

## Tryparedoxin peroxidase

Tryparedoxin peroxidase (TryP) is a principal enzyme that provides parasites with antioxidant defence through the detoxification of harmful peroxides (Flohé *et al*., 2003; Iyer *et al*., 2008). Since the Sb treatment is largely associated with the accumulation of deadly ROS in *Leishmania*, the enhanced expression of these proteins envisages the role of antioxidant defence and detoxification in the emergence of resistance (Fig. 2) (Wyllie *et al*., 2004; Mandal *et al*., 2007).

In *Leishmania*, the Sb stress elevated the TryP expression in both cytosol and mitochondria; however, the cytosolic expression was more remarkable than the mitochondrial counterpart (Wyllie *et al*., 2008; Das *et al*., 2018). The overexpression of TryP resulted in decreased Sb(III) sensitivity in *Leishmania*, while the overexpression of the enzymatically inactive form failed to bring about resistance, which corroborates the fact that TryP-mediated Sb resistance was independent of sequestration or membrane efflux of Sb(III) (Wyllie *et al*., 2008). A several-fold augmented TryP protein levels were reported in Sb-resistant *Leishmania,* accompanied by activated H_2_O_2_ metabolism as well as increased tolerance to exogenous H_2_O_2_ than the respective sensitive or parental lines (Andrade and Murta, 2014). Moreover, the elevation of TryP was also accompanied by a significant (>2 times average) TR expression in the resistant extracts, with an emphasis on the synergistic interactions of proteins involved in thiol-metabolism in gaining Sb resistance (Nateghi-Rostami *et al*., 2022). A 3 times TryP boost was observed in Sb-resistant *L. tarentolae* compared to that observed in lysates of revertant, along with a positive correlation to the subsequent peroxidase activity (Wyllie *et al*., 2008). In addition, a comparative proteomic analysis has revealed a highly abundant TryP expression in Sb(III)-resistant *L. braziliensis* and *L. infantum chagasi* cells (Matrangolo *et al*., 2013). In contrast, the incorporation of TryP through transfection neither exhibited significant gene expression nor tolerance of oxidative stress in *L. infantum* compared to the parental lines (Andrade and Murta, 2014).

## Heat shock proteins

The chemical inhibition of heat shock protein (HSP) was found to be a strategy to produce antileishmanial drugs (Das *et al*., 2020). Heat shock protein-83 (HSP83) and heat shock protein-70 (HSP70) are involved in antileishmanial drug-mediated programmed cell death by interfering with mitochondrial membrane potential (Vergnes *et al*., 2007).

Matrangolo *et al*. demonstrated prominent expression profiles of HSP83, heat shock protein-60 (HSP60), and HSP70 in the resistant cell lines of both *L. braziliensis* and *L. infantum chagasi*, particularly the HSP70 equivalents, having boosted in both the cytoplasm and the mitochondria of the *L. infantum chagasi* lines (Matrangolo *et al*., 2013). HSP70 and HSP83 were reported to be overexpressed in the membrane-enriched fraction of the SAG-resistant *L. donovani* clinical isolates (Kumar *et al*., 2010), and in the meantime, the increased expression pattern of HSP60 and HSP70 has been observed in relation to Sb-resistant mechanisms in *L. (Viannia) panamensis* (Peláez *et al*., 2012). The substantial abundance of HSP70 in *in vitro* selected Sb-resistant *Leishmania* of both amastigotes and promastigotes, and the corresponding revertant cells in the absence of drug pressure, could be suggestive of the stability and functional significance of this protein in Sb-resistant *Leishmania* (Brochu *et al*., 2004). Inversely, the Sb sensitivity was aligned with the downregulation of HSP83 in *L. donovani* (Kumar *et al*., 2012). In addition, the CL caused by naturally Sb-resistant *L. tropica* was associated with HSP70 differential expression (Özbilgin *et al*., 2021). The *L. infantum* promastigotes were superior to *L. tarentolae* in acquiring Sb(III) defence through robust HSP70 expression (Brochu *et al*., 2004). Nonetheless, a study proposed HSP70 to have only a 75% success rate as an effective candidate prediction model for determining Sb resistance of CL clinical cases caused by *L. braziliensis* (Torres *et al*., 2013).

However, a few of the findings possibly disprove the HSP-mediated Sb resistance in *Leishmania* as well as impose limitations on the suitability of this gene in predicting resistance. For instance, HSP83 was upregulated only in 40% of clinically resistant *L. donovani* lines compared to the LdAG83 reference strain (Kumar *et al*., 2012), and also the transfection of the HSP70 gene was unable to circumvent the Sb effect and confer direct resistance towards Sb (Brochu *et al*., 2004).

## Other important genes with elevated expression in Sb resistance

Only a few studies have been conducted on histone modification and gene regulation in trypanosomatids. There are 2 types of histones in *Leishmania*; core histones like H2A, H2B, H3, H4, and linker histone H1 (Soto *et al*., 1992, 1997; Fasel *et al*., 1993; Martínez *et al*., 2002). Histone expression in *Leishmania* has been implicated as a coupled mechanism to DNA replication that affects the level of translation *via* post-transcriptional mechanisms (Abanades *et al*., 2009), and a few studies have discussed the histone expression in Sb-resistant *Leishmania*. However, so far, histone expression has not been characterized as a direct contributor to drug resistance, but it may have a function in the epigenetic programming of resistance genes in *Leishmania*. Apart from that, histone epigenetic markers were essential for the survival of *L. major* (Anderson *et al*., 2013; Afrin *et al*., 2019). Meanwhile, the studies aiming to explore the correlation between histone function and antimonial drug resistance are not well established yet.

Overexpression of H2A into *L. donovani* conferred decreased Sb susceptibility against not only SAG but also developed resistance against amphotericin-B and miltefosine (Singh *et al*., 2010). H1 was found to have elevated expression in 9 out of 10 Sb-resistant *L. donovani* isolates, and H2A or H4 were upregulated in 50% of the SAG-resistant *L. donovani* clinical strains, however, it was found that there was a >2-fold downregulation in all the sensitive lines compared to the LdAG83-sensitive reference line (Kumar *et al*., 2012). Moreover, H1 and H2A showed protein level upregulation in SAG-resistant *L. donovani*, kala-azar (Singh *et al*., 2010). Based on the present data, the histone modification could be a potential activator of the resistant genes but has to be further characterized to reveal its exact molecular relationship to Sb resistance.

The novel Sb-resistant markers, ARM56 and ARM58 were significantly elevated in resistant *Leishmania*, although with unknown function or molecular mechanisms in this regard. ARM proteins contain conserved domains with hydrophobic amino acids and form transmembrane structures; particularly, ARM56 and ARM58 are located as a subtelomeric cluster in chromosome 34 and their co-expression has been implicated in Sb resistance (Nevado *et al*., 2016). Especially, the overexpression of ARM58 was found to minimize the Sb effect by reducing its accumulation in *Leishmania* cells by at least 70% (Schäfer *et al*., 2014). Most importantly, following *in vitro* selection for Sb(III), the ARM58 mRNA level exhibited an 800% boost compared to the wild-type and was also conferred protection on amastigotes as well as promastigotes against both Sb(III) and Sb(V) (Nühs *et al*., 2014). There was a clear correlation between Sb resistance and ARM56/ARM58 expression, whereas elevation of ARM58 was identified as an exclusive feature in Sb-resistant field isolates, hence urges for extensive studies before validating this protein as an Sb-resistant marker (Rugani *et al*., 2019).

Another protein, PRP1, belongs to the ABC transporter superfamily and was initially considered to confer cross-resistance towards the antimony trichloride but not Sb(V) (Coelho *et al*., 2003); nonetheless, a recent finding illustrated a significantly elevated expression in SSG-resistant *L. tropica* and *L. donovani* resulting in more than 4-fold expression (Khanra *et al*., 2022).

Parasite surface antigen-2 was consistently augmented in both the transcriptional and translational expression in SAG-resistant *L. donovani* clinical isolates resulting in more than 1.5-fold higher expression than the sensitive. Moreover, the overexpression of this protein could transform the sensitive strains to resistance with a decline of their Sb susceptibility level in >12-fold (Bhandari *et al*., 2013). Arsenate reductase-2 was a 3–4-fold expression in VL SSG-resistant *L. donovani,* and *L. tropica* compared to the sensitive reference strain AG83 (Khanra *et al*., 2022).

## Conclusions

Sb resistance in *Leishmania* is mainly achieved by the expression modification of Sb transporter genes, Sb-reducing enzymes, and thiol-synthesizing enzymes. Of the genes studied in the present review, MRPA, *γ*-GCS, ODC, TR, TDR1, TryP, and HSP illustrated a general likelihood of upregulation, while AQP1 and MAPK showed a tendency to downregulate in the Sb-resistant *Leishmania* and vice versa, therefore, the resistance may have been orchestrated by the functional relevance of genes. Likewise, the relative gene expression in Sb resistance can exhibit similarities among different resistant isolates, but there are chances for deviations to be made from the mostly accepted phenomena. The reason for the presence of inconsistencies of protein functions may be due to molecular adaptations like polymorphism and post-translational modifications. Altogether, the gene expression-based confirmation of Sb resistance in *Leishmania* by examining the upregulation or downregulation of a particular gene compared to a control will be useful in scientific studies to investigate the underlying biology; however, its application in patient diagnosis of clinical resistance may not be reliable due to possible false-positive or false-negative results and in some cases, the inadequacy of research work. Nonetheless, it is possible to minimize the potential misinterpretations provided that multiple resistance gene expressions are included in the analysis and if their relative expressions are highly significant. The current review circumscribed the Sb resistance-related gene expression to facilitate future research that will fulfil the unmet need for the detection of biomarkers for Sb-resistant leishmaniasis, which remains an obvious need to achieve effective disease control and elimination.

## Supporting information

Madusanka et al. supplementary material Madusanka et al. supplementary material

## Data Availability

There is no extra data for this paper.

## Supplementary material

The supplementary material for this article can be found at https://doi.org/10.1017/S0031182023001129
